# Thromboprophylaxis for Patients with High-risk Atrial Fibrillation and Flutter Discharged from the Emergency Department

**DOI:** 10.5811/westjem.2017.9.35671

**Published:** 2018-02-12

**Authors:** David R. Vinson, E. Margaret Warton, Dustin G. Mark, Dustin W. Ballard, Mary E. Reed, Uli K. Chettipally, Nimmie Singh, Sean Z. Bouvet, Bory Kea, Patricia C. Ramos, David S. Glaser, Alan S. Go

**Affiliations:** * The Permanente Medical Group, Oakland, California; † Kaiser Permanente, Division of Research, Oakland, California; ‡ Kaiser Permanente Sacramento Medical Center, Department of Emergency Medicine, Sacramento, California; § Kaiser Permanente Oakland Medical Center, Department of Emergency Medicine, Oakland, California; ¶ Kaiser Permanente San Rafael Medical Center, Department of Emergency Medicine, San Rafael, California; || Kaiser Permanente South San Francisco Medical Center, Department of Emergency Medicine, San Francisco, California; # Mercy Redding Family Practice Residency Program, Redding, California; ** Kaiser Permanente Walnut Creek Medical Center, Department of Emergency Medicine, Walnut Creek, California; †† Oregon Health and Science University, Department of Emergency Medicine, Portland, Oregon; ‡‡ Kaiser Permanente Sunnyside Medical Center, Northwest Permanente Physicians and Surgeons, Department of Emergency Medicine, Portland, Oregon; * The Permanente Medical Group, Oakland, California; † Kaiser Permanente, Division of Research, Oakland, California; §§ Sisters of Charity of Leavenworth St. Joseph Hospital, Department of Emergency Medicine, Denver, Colorado; ¶¶ University of California, San Francisco, Departments of Epidemiology, Biostatistics, and Medicine, San Francisco, California; || Stanford University School of Medicine, Department of Health Research and Policy, Palo Alto, California

## Abstract

**Introduction:**

Many patients with atrial fibrillation or atrial flutter (AF/FL) who are high risk for ischemic stroke are not receiving evidence-based thromboprophylaxis. We examined anticoagulant prescribing within 30 days of receiving dysrhythmia care for non-valvular AF/FL in the emergency department (ED).

**Methods:**

This prospective study included non-anticoagulated adults at high risk for ischemic stroke (ATRIA score ≥7) who received emergency AF/FL care and were discharged home from seven community EDs between May 2011 and August 2012. We characterized oral anticoagulant prescribing patterns and identified predictors of receiving anticoagulants within 30 days of the index ED visit. We also describe documented reasons for withholding anticoagulation.

**Results:**

Of 312 eligible patients, 128 (41.0%) were prescribed anticoagulation at ED discharge or within 30 days. Independent predictors of anticoagulation included age (adjusted odds ratio [aOR] 0.89 per year, 95% confidence interval [CI] 0.82–0.96); ED cardiology consultation (aOR 1.89, 95% CI [1.10–3.23]); and failure of sinus restoration by time of ED discharge (aOR 2.65, 95% CI [1.35–5.21]). Reasons for withholding anticoagulation at ED discharge were documented in 139 of 227 cases (61.2%), the most common of which were deferring the shared decision-making process to the patient’s outpatient provider, perceived bleeding risk, patient refusal, and restoration of sinus rhythm.

**Conclusion:**

Approximately 40% of non-anticoagulated AF/FL patients at high risk for stroke who presented for emergency dysrhythmia care were prescribed anticoagulation within 30 days. Physicians were less likely to anticoagulate older patients and those with ED sinus restoration. Opportunities exist to improve rates of thromboprophylaxis in this high-risk population.

## INTRODUCTION

Atrial fibrillation (AF) and atrial flutter (AFL) independently increase the risk of ischemic stroke five-fold and account for an estimated 15% of ischemic strokes. 1 For this reason, stroke prevention is one of the leading management objectives in the long-term care of patients with AF or AFL (AF/FL), regardless of rhythm duration or permanence. 2–5 Validated thromboembolism risk scores exist to help readily identify the high-risk AF/FL population that would benefit from long-term anticoagulation. 6–9 Nevertheless, underuse of thromboprophylaxis persists nationally and internationally, in large measure because physicians incorrectly assess levels of risks and benefits. 10–19

Non-anticoagulated patients with AF/FL commonly seek rhythm-related care in the emergency department (ED). 15, 20–22 AF patients who present for emergency care have a higher incidence of stroke and death than patients seen in other venues. 23 In some settings, more than half of AF patients discharged from the ED fail to achieve outpatient follow-up within 90 days of discharge, regardless of insurance status. 24 In such cases, an ED visit may provide a critical opportunity for a stroke-prevention intervention. Such encounters may also serve as a sentinel event for those at high risk for stroke, facilitating important changes in their health behavior. 25–30 Physicians can seize on such teachable moments to educate high-risk AF/FL patients on stroke risk and prevention and, when appropriate, to recommend or prescribe anticoagulation.^15^, ^31^

Initiating anticoagulation at the time of ED discharge for stroke-prone patients does not increase bleeding rates and contributes to decreased mortality. 32 Some patients, however, might prefer to have this shared decision-making conversation with a provider aware of their values and preferences, e.g., a primary care provider or cardiologist. 3 Nevertheless, emergency physicians (EP) are an important link in the chain of multi-specialty care coordination for the stroke-prone AF/FL population—whether they initiate the discussion of thromboprophylaxis or actually prescribe anticoagulation. 33,34

The initiation of thromboprophylaxis to ED patients with AF/FL at high risk for stroke has not been extensively studied. The literature that exists, however, demonstrates under-prescribing in countries around the world. 12, 15, 20, 35–37 The prescribing practices in U.S. community EDs, however, are not well understood.

We undertook a multicenter, prospective, observational study to evaluate the anticoagulation practice patterns of community EPs and short-term, post-ED care providers in the management of patients with non-valvular AF/FL considered at high risk for ischemic stroke. We also sought to identify factors influencing initiation of oral anticoagulation. We hypothesized that increasing age, lack of cardiology involvement in the patient’s ED care, and restoration of sinus rhythm before ED discharge would decrease the likelihood of receiving an oral anticoagulant prescription. Lastly, we reviewed the electronic health records of the patients discharged without anticoagulation to evaluate documented reasons for withholding anticoagulation and provision of educational material on AF/FL stroke risk and prevention.

**Population Health Research Capsule**

What do we already know about this issue?

Oral anticoagulation can reduce strokes by two-thirds in patients with non-valvular atrial fibrillation or flutter (AF/FL), yet many high-risk patients remain untreated.

What was the research question?

What is the incidence of anticoagulation initiation within 30 days of emergency AF/FL care for high stroke-risk patients?

What was the major finding of the study?

Only 41% of untreated high-risk patients received an anticoagulant prescription at ED discharge or in the following 30 days.

How does this improve population health?

Multidisciplinary efforts to reduce strokes in high-risk AF/FL patients will need to address physician misunderstandings of anticoagulation risks and benefits and improve patient education.

## METHODS

The study was approved by the Kaiser Permanente Northern California (KPNC) Institutional Review Board. Waiver of informed consent was obtained due to the observational nature of the study.

### Study Design and Setting

This study was a sub-analysis of a prospective observational study (TAFFY, Treatment of AF/FL in the emergencY department). 38 The source population was based within KPNC, a large integrated healthcare delivery system that provides comprehensive medical care for four million members across 21 medical centers. KPNC members represent approximately 33% of the population in areas served and are highly representative of the local surrounding and statewide population.

Emergency care was provided by emergency medicine residency-trained and board-certified (or board-prepared) EPs. During the study period (May 2011 to August 2012), the annual census of each of the seven EDs ranged from 25,000 to 78,000. No departmental policies were in place at the participating EDs to govern the short-term anticoagulation management of patients with AF/FL. Patient care was left to the discretion of the treating EPs.

All facilities had pharmacy services available around-the-clock for discharge medications and supplemental patient education. Oral anticoagulation medications in use within KPNC during the study period were warfarin and dabigatran, warfarin being the drug of choice at the time. Furthermore, each facility had its own pharmacy-managed, phone-based Outpatient Anticoagulation Service that managed outpatient warfarin use and provided close follow-up and monitoring of these patients, akin to similar programs in other KP regions in the U.S. 39, 40 The percent time in therapeutic range for the international normalized ratio during the study period varied by facility and ranged from 70% to 74%, calculated with a six-month look-back period using the Rosendaal linear interpolation method.^41^

### Selection of Participants

In the TAFFY study, adult (≥18 years) KPNC health plan members in the ED with electrocardiographically-confirmed non-valvular AF/FL were eligible for prospective enrollment if their atrial dysrhythmia fell into any one of these three categories: (1) symptomatic AF/FL; (2) AF/FL requiring ED treatment for rate or rhythm control; or (3) the first known electrocardiographically-documented episode of AF/FL (that is, newly diagnosed). Patients were ineligible if they were transferred in from another ED, were receiving only palliative comfort care, had an implanted cardiac pacemaker/defibrillator, or had been resuscitated from a cardiac arrest in the ED or just prior to arrival. The treating EPs enrolled patients via convenience sampling and were provided a small token of appreciation for their bedside data collection. No research assistants facilitated enrollment.

This anticoagulation study included TAFFY patients who were (1) not taking oral anticoagulants at the time of ED presentation; (2) at high risk for thromboembolic complications based on a validated thromboembolism risk score; and (3) discharged home directly from the ED. Only a patient’s first enrollment was included in this analysis. We used the validated Anticoagulation and Risk Factors in Atrial Fibrillation (ATRIA) stroke risk score (see below) to identify our AF/FL population at high risk for thromboembolism, as it has been shown to be more accurate than the CHADS_2_ or CHA_2_DS_2_-VASc stroke risk scores. 9, 42, 43

### Methods and Measurements

TAFFY variables collected prospectively at the time of patient care included presenting symptoms; characterization of the atrial dysrhythmia (AF, AFL, or both; new, first-time diagnosis; physician’s impression of clinical category [intermittent/recurrent; chronic/sustained, 24/7; unclear]; recent onset of rhythm-related symptoms [<48 hours]); comorbid diagnoses; ED management (rate reduction, attempted cardioversion); cardiology consultation; discharge rhythm and discharge pharmacotherapy. To minimize the effect that structured data collection might have on stroke prevention and to improve the odds of describing real-world behavior, the physician education material and data collection tool mentioned none of the following: hemorrhage risk, thromboembolic risk, risk scoring, indications for anticoagulation, post-ED follow-up care, or this study’s objectives and hypotheses. We undertook monthly manual chart review audits at each medical center to identify cases that were TAFFY-eligible but had not been enrolled to assess potential selection bias between the enrolled and missed-eligible populations.

After completion of the enrollment period, we extracted additional demographic and clinical variables from the health system’s comprehensive integrated electronic health record. These included additional patient characteristics and oral anticoagulation prescription, prescriber, and outpatient follow-up within 30 days of ED discharge.

### Stroke Risk

We retrospectively calculated the ATRIA stroke risk score from structured data in the comprehensive electronic health record using definitions of score variables from the original derivation and validation studies. 9 The ATRIA stroke risk score uses weighted scoring based on points assigned for age, prior history of ischemic stroke, female gender, diabetes mellitus, chronic heart failure, hypertension, known proteinuria, and estimated glomerular filtration rate <45 ml/min/1.73 m^2^ or end-stage renal disease treated with dialysis or kidney transplant (Table 1). 9 Patients with an ATRIA score ≥7 were categorized as high risk.

### Bleeding Risk

We characterized predicted bleeding risk using a modified HAS-BLED score. 44, 45 HAS-BLED is an acronym for hypertension (uncontrolled, >160 mmHg systolic), abnormal renal/liver function (one point for presence of renal or liver impairment, maximum two points), stroke (previous history, particularly lacunar), bleeding history or predisposition (anemia), labile international normalized ratio (INR) (i.e., therapeutic time in range <60%), elderly (>65 years), drugs or alcohol (antiplatelet agents, nonsteroidal anti-inflammatory drugs; one point for drugs plus one point for alcohol excess, maximum two points). Patients with a HAS-BLED risk score of ≥3 were deemed at high risk for bleeding. We modified the HAS-BLED score slightly to accommodate retrospective identification of structured variables in the electronic health record (see Table A.1, in the online appendix). 46

### Manual Chart Review

We undertook a structured manual chart review of the ED provider notes and discharge instructions for patients discharged home without anticoagulation. We identified physician and patient reasons for not starting anticoagulation. We noted also if patient education material on AF/FL, which included a mention of the association of these dysrhythmias with thromboembolic events, was included in the printed discharge instructions. We entered our findings directly into a standardized electronic data collection instrument, modified to its final form after pilot testing. The physician abstractors received training on data collection methods and were not blinded to the study objectives. The principal investigator answered and arbitrated coding questions until consensus was formed, and monitored data collection activities. A random sample of cases (10%) was selected for independent review to assess documentation of reasons for anticoagulation non-prescribing and presence of patient education material on AF/FL at ED discharge.

### Outcomes

Our primary outcome was a new oral anticoagulation prescription ordered by either the EP at the index ED discharge or a subsequent provider within 30 days of the index ED visit. We captured all prescription orders of anticoagulants in our comprehensive pharmacy databases. Our secondary outcomes were the reason for withholding anticoagulation documented in the EP’s note for patients discharged without anticoagulation and inclusion of AF/FL patient education material in the printed discharge instructions.

### Statistical Analysis

We conducted all analyses using SAS statistical software, version 9.31 (Cary, N.C.). A two-tailed p value of less than 0.05 was considered significant. We compared characteristics between those enrolled and not enrolled in the study, as well as groups with and without anticoagulation initiation in the study, using chi-square tests for categorical variables and t-tests or Wilcoxon rank-sum tests for continuous variables. Univariate logistic regression models for the outcome of receipt of anticoagulants within 30 days of the index AF/FL visit identified possible predictors. After running a fully saturated adjusted model, we retained predictors that were significant (p<0.05) along with age, gender, and race/ethnicity to generate a final multivariable model estimating odds ratios and 95% confidence intervals. We measured interrater reliability for chart-reviewed variables using both percent agreement and an unweighted kappa statistic.

## RESULTS

Among 2,849 identified eligible patients, 1,980 (69.5%) were enrolled by the treating physicians in the parent TAFFY study. Enrolled and non-enrolled patients were comparable in terms of age, gender, comorbidity, and stroke risk scores, except that enrolled patients were more likely to have had a history of prior diagnosed AF/FL (see Table A.2, in the online appendix). For the present analysis, we excluded 906 enrolled patients (45.8%) who were not discharged home directly from the ED or were not KP health plan members at enrollment, 252 patients (23.5%) who were already taking anticoagulation therapy and 510 patients (62.0%) who were not high risk for thromboembolism (ATRIA score <7) (Figure). The remaining 312 AF/FL patients constituted our study cohort. While selected for the study based on their ATRIA score, all study patients were also found to be high risk using the CHA_2_DS_2_-VASc score (≥2 points). 7 Overall, median age was 80 years (interquartile range, 76 to 85), and 201 (64.4%) cohort members were women.

Oral anticoagulants were prescribed to 128 patients (41.0%) within 30 days of the index ED visit, with 85 patients (27.2%) receiving a new anticoagulant prescription at the time of ED discharge and the remaining 43 patients (13.7%) in the following 30 days. In this sample, warfarin was the only oral anticoagulant prescribed. During the post ED-discharge period, the specialty of the physician prescribing anticoagulation included outpatient internal medicine (n=30), cardiology (n=6), hospital medicine (n=4), and emergency medicine (n=3). Among the 227 patients who left the ED without an oral anticoagulant prescription, 195 (85.9%) had an in-person or telephone encounter with a primary care provider or cardiologist within 30 days.

Forty-three patients (13.8%) were discharged home only on antiplatelet medications: seven were advised to continue their daily aspirin and 36 were prescribed (or advised to begin) new daily antiplatelet agents at the time of discharge (35 aspirin and one clopidogrel).

Characteristics of the cohort stratified by anticoagulation initiation are described in Table 2.

Variables independently associated with increased odds of anticoagulation initiation included younger age, new diagnosis of AF/FL, symptom onset >48 hours prior to evaluation, EP assessment of rhythm pattern as intermittent (not unremitting), receipt of cardiology consultation in the ED, and failure of sinus restoration by time of ED discharge (Table 3).

Among the 227 patients discharged home from the ED without anticoagulation, 139 patients (61.2%) had one or more reasons documented for withholding anticoagulation. These were categorized as physician concerns and patient concerns (Table 4). The leading physician reasons for withholding anticoagulation were concerns about elevated bleeding risk (including fall risk), deferring the decision to an outpatient provider, and the perception that the restoration of sinus rhythm had significantly reduced or eliminated stroke risk. The leading patient reasons for declining anticoagulation were a preference to continue the discussion of anticoagulation with their outpatient provider and simple refusal, not otherwise specified. Deferring the shared decision-making process to the patient’s outpatient provider was the leading reason for withholding anticoagulation when combining physician and patient concerns (43/227; 18.9%).

One hundred thirty-seven (60.3%) patients were given patient education material on AF/FL in their discharge instructions. The three versions of material used by the EPs each included one sentence about the general association between AF/FL and thromboembolic events. The material was not personalized, however, and did not quantify the patient’s specific risk (e.g., 4.0% annual stroke risk), nor even mention broader thromboembolic risk categories (low, moderate, high), nor discuss the benefits and risks of stroke prevention therapy.

Using an online random number generator (random.org; Randomness and Integrity Services Ltd., Dublin, Ireland), we identified 23 cases for review by a second abstractor. Percent agreement was the same for presence of both documented reason for non-prescribing and provision of patient education material (22/23; 96.5%). The kappa statistic was 0.91 for each variable.

## DISCUSSION

In this multicenter, prospective cohort of non-anticoagulated AF/FL patients at high thromboembolic risk discharged home from the ED, we found that approximately 40% were prescribed oral anticoagulation within 30 days. Furthermore, we observed that younger age, selected rhythm-related characteristics in the ED, and receipt of cardiology consultation were strongly associated with receiving anticoagulation.

About 60% of patients discharged home from the ED without anticoagulation had a reason documented in their electronic health record, a relatively high percentage of documentation compared with a recent, large, inpatient registry. 10 The principal reason for non-prescribing in our study was deferring the shared decision-making process to the patient’s outpatient provider (18.9%). Such reasoning is sensible in a setting like ours where patients have ready access to their outpatient physicians and 30-day follow-up is common. 47 Our percentage of deferral was higher than in a similar study of ED anticoagulation prescribing for high-risk AF/FL in Spain (5.6%), though, like our study population, all of their patients also had health coverage.^20^ Other leading documented reasons included a perception of increased bleeding risk (e.g., falls) and a perception of reduced stroke risk (e.g., when paroxysmal AF/FL reverted to sinus rhythm prior to ED discharge).

### ED Anticoagulation Initiation in the Literature

The incidence of oral anticoagulation initiation for AF/FL patients at high risk for ischemic stroke who are discharged home from the ED has not been well described. Reports range widely, from approximately 10% to 50%. The calculation also varies depending on whether stroke-prone AF/FL patients deemed ineligible for anticoagulation are included in the denominator. A large, 124-center study from Spain by Coll-Vinent et al. in 2011 demonstrated that anticoagulation was initiated at the time of home discharge to 193 of 453 high-risk AF patients (44%), higher than our 27%.^20^ The case mix in this study was similar to ours in that patients with all categories of AF were included (e.g., first episode, paroxysmal, persistent, and permanent), but was different in that they excluded patients thought ineligible for anticoagulation, something our study design did not allow. This difference might explain in part why their incidence of initiation was higher than what we observed. A more recent, 62-center Spanish study from the same investigators reported a similarly high incidence of de novo anticoagulation prescribing on ED discharge. 32 Two hospitals with the University of British Columbia, Canada, have reported a high baseline incidence (49%; 51/105) of appropriate anticoagulation initiation at ED discharge for high-risk AF/FL patients. As with the Spanish study above, these investigators had excluded ineligible patients.^48^

Other studies have reported lower incidences of anticoagulation initiation. A retrospective cohort study undertaken in 2008 in eight Canadian EDs observed thromboprophylaxis initiation in 21 of 210 patients (18%) with recent-onset AF/FL who were discharged home. 37 A more recent prospective study by Stiell et al. described the treatment of patients with recent-onset AF at six academic Canadian EDs from 2010 to 2012 and found slightly lower rates of untreated high-risk patients leaving the ED with a new anticoagulation prescription (approximately 11%).^12^ In a retrospective study of two academic Canadian EDs, Scheuermeyer et al reported that 27% (41/151) of high-risk AF/FL patients were begun on appropriate stroke prevention medications at discharge, and documentation of reasons for withholding thromboprophylaxis was noted in an additional 21 patients.^15^

### Patient Age

Our finding that older patients with high-risk AF/FL were less likely to receive an oral anticoagulant prescription than their younger counterparts is consistent with studies demonstrating under-treatment both in the ED^20^,^49^ and in other settings.^50^ Thromboprophylaxis is less commonly prescribed to patients over 75 years of age, even though this population likely benefits the most given their higher absolute risk of ischemic stroke compared with intracranial hemorrhage or life-threatening extracranial hemorrhage.^51^ Physicians often acknowledge their hesitancy to initiate anticoagulation in the elderly and very elderly,^52^ given that these patients often have a high comorbidity burden, associated cognitive disorders and polypharmacy-related challenges. Despite these concerns, there is often a misunderstanding about the net clinical benefit associated with oral anticoagulation in the elderly.^51^,^53^

Physicians often cite perceived bleeding risk as a primary reason for withholding anticoagulation for AF/FL patients, a finding we also observed. 32,52 Physicians overestimate the risk of intracranial bleeding in patients with high risk for falls. However, there is evidence that patients with AF would need to fall repeatedly throughout the year before the risk of intracranial hemorrhage would outweigh the net benefits of stroke-prevention from anticoagulation. 54 Of interest, a patient’s predicted hemorrhage risk, as measured by the HAS-BLED score, was associated with less anticoagulation prescribing in our population but was of borderline statistical significance (Table 3). The evidence suggests that a high-risk HAS-BLED score per se is not a reason to withhold anticoagulation that is otherwise indicated. 2, 55 In most patients with elevated bleeding risks, the magnitude of gain from stroke reduction far outweighs the small risk of serious bleeding. 56 Bleeding risk scores are best used to identify patients in need of closer follow-up, particularly to address reversible risk factors such as uncontrolled hypertension, concomitant use of non-steroidal anti-inflammatory medications, and excess alcohol. 2, 55

### Rhythm-related Characteristics

Our study found that several rhythm-related characteristics were strongly associated with the likelihood of receiving oral anticoagulation at or shortly after an ED visit for AF/FL. For example, we noted that patients who reverted to sinus rhythm before ED discharge were less likely to receive a prescription for anticoagulation. As indicated from the reasons documented for withholding anticoagulation, EPs significantly varied their estimation of a patient’s ischemic stroke risk based on the persistence of AF/FL during the ED stay. Lower rates of anticoagulation also have been seen in patients with paroxysmal AF in other practice settings. 13, 20 Most recently, an analysis of the American College of Cardiology PINNACLE Registry found that patients with paroxysmal AF considered at a moderate to high risk of ischemic stroke were less likely to be prescribed oral anticoagulant therapy and more likely to be prescribed less effective or no therapy for thromboembolism prevention than those with non-paroxysmal AF. 57

Compared with patients with persistent or permanent AF, those with paroxysmal AF have less frequent and less prolonged episodes of AF (that is, a lower overall “AF burden”), which correlates with a lower incidence of thromboembolism. 58–60 Yet the reduction in stroke risk is not sufficient to lessen the need for thromboprophylaxis. 3, 61 Importantly, consensus-based clinical practice guidelines do not vary their recommendations for thromboprophylaxis based on type of AF, nor do validated stroke risk scores alter their prognosis based on paroxysmal or non-paroxysmal rhythm. 3, 7, 9,62

We also found that physicians were less likely to initiate anticoagulation in patients with a history of prior AF/FL and in those whose atrial dysrhythmia was thought by the EP to be chronic and unremitting. This might seem counterintuitive given our finding that patients who left the ED still in AF/FL were more likely to receive thromboprophylaxis. It is possible, however, that ED patients at high risk for stroke with known recurrent or chronic AF/FL had already been advised about anticoagulation options before their index ED visit and previously declined or discontinued anticoagulation in the distant past. Some have attributed this behavior to “clinical inertia,” the hesitancy of physicians to alter the current pattern of care initiated by other providers. 63 Nonetheless, further exploration is needed to clarify the underlying reasons for these observations. With today’s expanded pharmacopeia for AF/FL stroke prevention, patients who had declined or discontinued warfarin in the past may be open to consider a direct oral anticoagulant, given the several patient-oriented advantages of this class of medications. 62, 64–66

### ED Cardiology Consultation

One novel finding of our study is that EPs were more likely to initiate anticoagulation when consulting cardiology. The reason for this may be multifactorial. Certain patients may have a clinical profile that leads to both cardiology consultation and thromboprophylaxis, or perhaps EPs who consult cardiology are more apt to initiate anticoagulation independent of the consultation. The more likely reason, however, is that cardiologists asked to advise on any facet of ED AF/FL care may raise the question of stroke risk and recommend thromboprophylaxis when indicated. 67 Others have shown that cardiology involvement in the outpatient setting improves rates of stroke prevention treatment in AF patients. The TREAT-AF study found that outpatient cardiology care compared with primary care was associated with higher rates of anticoagulation of AF patients. 68 Anticoagulation rates increase even when a primary care provider referred their AF/FL patients to see a cardiologist but maintained patient oversight themselves. 67

### Post-ED Outpatient Follow-up

The benefits of multispecialty collaboration were seen not just during the patients’ ED stay. Of those who were prescribed oral anticoagulation in this study, more than one quarter were given thromboprophylaxis in the outpatient setting, either in the primary care or cardiology clinics. The importance of post-ED follow-up for AF/FL patients at high risk for thromboembolism is also seen by the number of EPs and patients in our study who deferred the anticoagulation decision to allow a fuller discussion of thromboprophylaxis with an outpatient provider (nearly one in five).

Deferring the initiation of anticoagulation in high-risk ED patients, however, may not be without risk. In some settings, a significant proportion of AF patients discharged home from the ED failed to achieve outpatient follow-up in the subsequent 90 days, regardless of insurance status. 24 Moreover, compared with patients who leave the ED with an anticoagulant prescription in hand, those who wait to consult an outpatient provider about stroke prevention have been shown to have a significantly lower frequency of long-term anticoagulation use (76% vs. 36% at one year) and a significant delay in initiation among those eventually treated (mean start time of 205 days following index ED discharge). 69 When referring patients to outpatient providers for this critical decision, the EP can facilitate anticoagulation initiation by several means: (1) introducing stroke prevention to their AF/FL patients and beginning (or continuing) the educational and shared decision-making process; (2) including stroke prevention material in the patient’s discharge instructions; (3) recommending (or even securing) a timely follow-up appointment; and (4) notifying the outpatient provider that stroke prevention may be indicated and that patient education was begun prior to ED discharge.

### Opportunities to Improve Care

Our results highlight opportunities for improvement in care. Patients seeking emergency care for their AF/FL may be more open to health-promoting behavioral changes, as has been observed with other medical conditions. 25–30 Initiating stroke-prevention therapy at the time of ED discharge has been shown to be safe and associated with a mortality reduction.^32^ Not all EPs, however, see it as their role to initiate anticoagulation when indicated for AF/FL patients.^15^,^31^ Nevertheless, EPs can still play a key role in promoting stroke prevention by risk-stratifying their AF/FL patients, broaching the topic with high-risk patients, adding personalized stroke-risk educational material to the discharge instructions, and encouraging high-risk patients to continue the shared decision-making conversation about thromboprophylaxis with their outpatient provider.

The results of this study raise questions about other ways to increase evidence-based anticoagulation. We identified certain physician misunderstandings that, if corrected, could increase anticoagulation of stroke-prone patients with AF/FL. Physician education should emphasize that patients with AF/FL at high risk for thromboembolism warrant stroke prevention even if their rhythm type is paroxysmal. 3, 7, 9 Also, antiplatelet agents do not provide sufficient protection against ischemic stroke in patients with high-risk AF/FL, though this is commonly believed.^20^,^70^ We observed that about one in eight high-risk AF/FL patients were given or continued on aspirin instead of oral anticoagulation, a high percentage, but lower than that found in a large cardiology clinic-based population of AF patients at moderate to high risk of stroke.^71^ Unfortunately, we were not able to distinguish when aspirin was advised as though it were sufficient stroke prevention from cases where the patient refused anticoagulation and was recommended aspirin instead.

Recent U.S. guidelines suggest a very limited role for aspirin in selected AF/FL patients (i.e., those with low predicted risk of stroke); 3 data supporting the use of aspirin monotherapy in patients at high risk of stroke are poor, and there are reports that it may even increase the risk of ischemic stroke in elderly patients.^72^ Aspirin is also not safer than oral anticoagulation in patients over 80 years of age with regard to serious bleeding. 73 Recent guidelines recommend that aspirin monotherapy should not be used as stroke prevention in AF/FL with the exception of patients who refuse any form of oral anticoagulation and cannot tolerate a combination of aspirin and clopidogrel. 2, 74

Though education about these misunderstandings will be vital, education alone may ultimately have little impact on changing physician behavior. 75,76 Several academic medical centers have improved oral anticoagulation rates in stroke-prone AF patients by referring them to an accessible outpatient AF clinic. 77,78 Another recommended approach is the provision of electronic clinical decision support to help physicians in their care for AF/FL patients. 14 To facilitate AF/FL thromboprophylaxis, such a system could calculate a patient’s predicted stroke and bleeding risk scores simultaneously at the point of care and provide patient-specific recommendations for treatment. Results of various clinical decision support systems have been mixed. 63, 79–81 The more effective systems have taken a multimodal approach. The Anticoagulant Programme East London, for example, showed improvement in appropriate anticoagulation of outpatients with AF by a combined program of education around agreed-upon guidelines with computer aids to facilitate decision-making as well as patient-specific review and feedback of locally identifiable results.^81^ Some clinical researchers are sharing their electronic clinical decision support tools for AF stroke prevention with patients and have found that mobile health technology improved patient knowledge, drug adherence, anticoagulant satisfaction, and quality of life.^82^

Electronic clinical decision support tools have had success in the ED setting when combined with a strong promotional program and could be readily adapted for use in patients with AF/FL.^83^–^86^ A multidisciplinary team at the University of British Columbia designed such an electronic clinical care pathway for ED patients with uncomplicated AF/FL.^48^ The pathway included a care map, decision aids, medication orders, management suggestions, and electronic consultation or referral documents, embedded in the computerized physician order entry and integrated electronic health record. Implementation was preceded and accompanied by a standardized educational and promotional program. The pathway increased the incidence of anticoagulation initiation on discharge for high-risk patients by 20.6 percentage points (from 48.6% to 70.2%).^48^

## LIMITATIONS

This study had several limitations. The study sample did not include all identified AF/FL patients; however, patient characteristics were highly similar between those who were and were not enrolled, so the impact of potential selection bias is likely limited. Our prospective data collection tool was designed to evaluate a wide range of care-related issues for AF/FL and was not focused on thromboprophylaxis (see above), but we cannot rule out the potential for a Hawthorne effect during the study period. The sample size was modest, which accounts for limited precision for certain associations, and we cannot rule out missing associations of smaller magnitude that may still be clinically relevant. We did not prospectively capture each patient’s relative contraindications to anticoagulation or their treatment preferences, which are two of the leading reasons physicians deviate from guideline recommendations for stroke prevention therapy. 87 This enlarged our denominator of anticoagulant-eligible patients and lowered our percentage of anticoagulant prescribing. We were able to identify some of these contraindications during our retrospective chart review, but these variables were incompletely documented.

This study focused on stroke prevention using warfarin, the only oral anticoagulant on the formulary in our health system until early 2014. Even with the recent availability of direct oral anticoagulants, physicians in our health system continue to initiate warfarin for AF/FL thromboprophylaxis: 40% of new oral anticoagulant prescriptions during the first quarter of 2017 for non-valvular AF/FL (with or without additional anticoagulation indications) across all 21 medical centers were for warfarin. Warfarin continues to be widely used for stroke prevention across North America, Europe, and around the world. 88 In fact, the European Society of Cardiology says it is reasonable to continue warfarin therapy in AF patients with a reassuring time in therapeutic range.^62^, ^89^ It is unclear whether the availability of newer agents will substantively alter physician overestimation of bleeding risk in older patients or underestimation of long-term stroke risk in patients with paroxysmal AF. Additional research will be needed to evaluate whether practice patterns of stroke prevention in AF/FL patients will change with use of direct oral anticoagulants. Studies suggest, however, that suboptimal AF thromboprophylaxis persists despite the availability of direct oral anticoagulants.^90^

Lastly, our study was conducted in a large integrated healthcare delivery system in California among insured patients who, on ED discharge, can receive close monitoring by our pharmacy-led Outpatient Anticoagulation Service and timely follow-up with their primary care providers. These integrated services may influence ED prescribing practices and may not be readily available to patients and providers in other healthcare systems. These distinctions of care may limit the generalizability of our results to other geographic locations and practice settings.

## CONCLUSION

In summary, we found that about 40% of non-anticoagulated patients with AF/FL at high risk for stroke who received rhythm-related care in the ED were prescribed evidence-based thromboprophylaxis in the ED or within 30 days. Younger age, ED cardiology consultation, and failure of sinus restoration at time of ED discharge increased the odds of anticoagulation initiation. Reasons for withholding anticoagulation included deferring the decision-making to the outpatient setting, as well as perceptions of high bleed risk (e.g., fall risk) and low stroke risk (e.g., paroxysmal AF/FL). Discharge instructions on AF/FL either lacked personalized stroke risk information or were absent altogether. Opportunities exist to improve stroke prevention interventions in this high-risk population. Multidisciplinary efforts to reduce strokes in high-risk AF/FL patients will need to address physician misunderstandings of anticoagulation risks and benefits and improve patient education.

## Supplementary Information



## Figures and Tables

**Figure:**
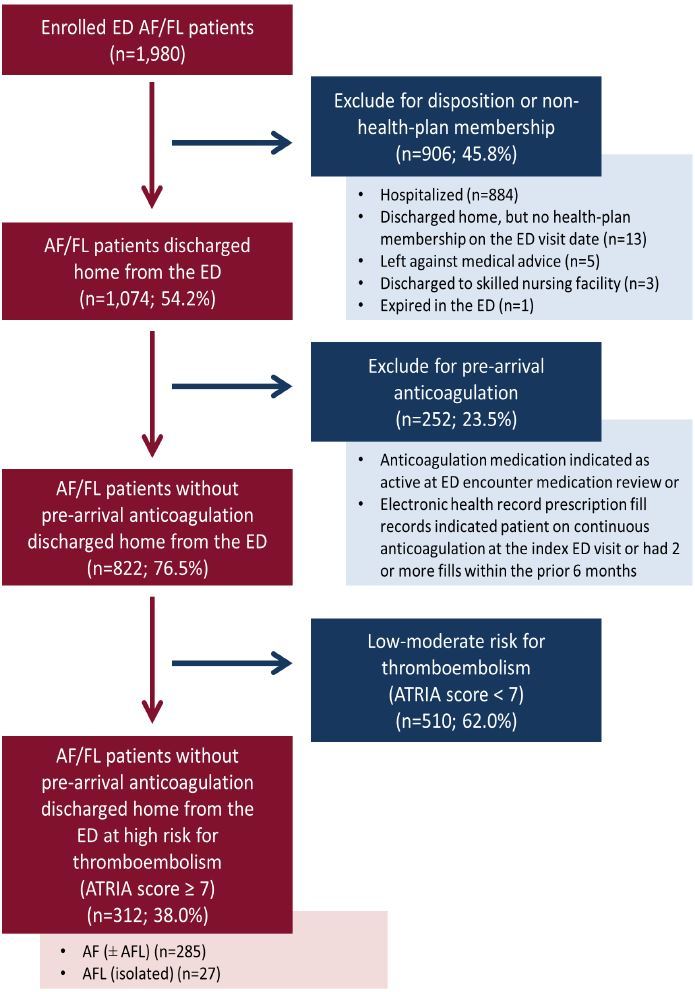
Patient flow of emergency department (ED) patients with eligible atrial fibrillation or flutter (AF/FL) enrolled in the TAFFY study *TAFFY*, Treatment of Atrial Fibrillation and Flutter in the emergencY department; *ATRIA*, Anticoagulation and Risk Factors in Atrial Fibrillation Study.

**Table 1:** ATRIA stroke risk score components and point assignment for adults with atrial fibrillation.^9^

Risk factor	Points assigned*
Age, yr	
≥85, with prior ischemic stroke	9
75 to 84, with prior ischemic stroke	7
65 to 74, with prior ischemic stroke	7
<65, with prior ischemic stroke	8
≥85, without prior ischemic stroke	6
75 to 84, without prior ischemic stroke	5
65 to 74, without prior ischemic stroke	3
Female gender	1
Diabetes mellitus	1
Chronic heart failure	1
Hypertension	1
Proteinuria	1
eGFR<45 ml/min/1.73 m^2^ or end-stage renal disease	1

*ATRIA,* Anticoagulation and Risk Factors in Atrial Fibrillation; *eGFR,* estimated glomerular filtration rate.

* A total point score for a given patient corresponds with the following risk classes: 0–5 points, low risk; 6 points, moderate risk; 7–15 points, high risk.

**Table 2:** Characteristics of atrial fibrillation and flutter patients at high risk for stroke who were discharged home from the emergency department, stratified by anticoagulation initiation.

Patient characteristics	Anticoagulation initiation in ED or within 30 days			

	Total (N=312)	Yes (n=128, 41.0%)	No (n=184, 59.0%)	P-value*
Age at ED visit, years				
Mean (SD)	80.4 (6.8)	78.5 (5.8)	81.8 (7.1)	<0.001
Categorical, n (%)				
65 to 74	48 (15.4)	24 (18.8)	24 (13.0)	0.17
≥75	264 (84.6)	104 (81.2)	160 (87.0)	
Female gender, n (%)	201 (64.4)	75 (58.6)	126 (68.5)	0.07
Race				0.29
White/European	262 (84.0)	105 (82.0)	157 (85.3)	
Asian/Pacific Islander	25 (8.0)	13 (10.2)	12 (6.5)	
Black/African American	16 (5.1)	7 (5.5)	9 (4.9)	
Native Hawaiian/other Pacific Islander	2 (0.6)	1 (0.8)	1 (0.5)	
Other/unknown	7 (2.24)	2 (1.6)	5 (2.7)	
Comorbidities and scores				
History of atrial fibrillation and flutter	137 (43.9)	37 (28.9)	100 (54.3)	<0.001
Hypertension	264 (84.6)	112 (87.5)	152 (82.6)	0.23
Proteinuria	168 (53.8)	69 (53.9)	99 (53.8)	0.99
Diabetes mellitus	83 (26.6)	44 (34.4)	39 (21.2)	0.01
Coronary heart disease	75 (24.0)	40 (31.3)	35 (19.0)	0.01
Estimated GFR <45 ml/min/1.73 m^2^ or end-stage renal disease	62 (19.9)	28 (21.9)	34 (18.5)	0.46
Chronic heart failure	44 (14.1)	18 (14.1)	26 (14.1)	0.99
Peripheral artery disease	13 (4.2)	8 (6.3)	5 (2.7)	0.13
Prior ischemic stroke	4 (1.3)	1 (0.8)	3 (1.6)	0.50
ATRIA study stroke risk score				
Mean (SD)	12.5 (3.8)	11.7 (3.3)	13.1 (4.0)	<0.001
Median (IQR)	11.5 (10–16)	11 (10–13)	12 (10.5–17)	
HAS-BLED hemorrhage risk score				
Mean (SD)	2.6 (1.4)	2.4 (1.3)	2.7 (1.4)	0.07
Median (IQR)	2.0 (2–3)	2.0 (2–3)	2.0 (2–4)	
Categorical, n (%)				
Low risk (<3)	179 (57.4)	83 (64.8)	96 (52.2)	0.03
High risk (≥3)	133 (42.6)	45 (35.2)	88 (47.8)	
Rhythm characteristics				
Diagnosis				
Atrial fibrillation (any)	285 (91.3)	110 (85.9)	175 (95.1)	<0.01
Atrial flutter (isolated)	27 (8.7)	18 (14.1)	9 (4.9)	
Recent-onset of rhythm-related symptoms (<48 hours)				
Yes	147 (47.1)	50 (39.1)	97 (52.7)	0.04
No	68 (21.8)	35 (27.3)	33 (17.9)	
Unclear	97 (31.1)	43 (33.6)	54 (29.3)	
Impression of clinical category				
Intermittent/recurrent	208 (66.7)	88 (68.7)	120 (65.2)	0.01
Chronic/sustained	41 (13.1)	9 (7.0)	32 (17.4)	
Unclear	63 (20.2)	31 (24.2)	32 (17.4)	
Sinus rhythm at discharge	140 (44.9)	48 (37.5)	92 (50.0)	0.03
ED cardiologist consultation	117 (37.5)	64 (50.0)	53 (28.8)	<0.001

*ED,* emergency department; *GFR*, glomerular filtration rate; *ATRIA*, Anticoagulation and Risk Factors in Atrial Fibrillation; *HAS-BLED*, Hypertension, Abnormal renal/liver function, Stroke, Bleeding history or predisposition, Labile international normalized ratio, Elderly (>65 years), Drugs or alcohol; *SD*, standard deviation; *IQR*, interquartile range.

* P-values from chi-square likelihood ratio tests for all categorical comparisons. For comparison of means, Student t-tests are reported.

**Table 3:** Association of variables with 30-day anticoagulation initiation for high-risk patients (ATRIA score ≥7) with atrial fibrillation and flutter discharged home from the emergency department

Variable	Anticoagulation initiation in ED or within 30 days			

	Univariate models		Multivariable model	
	
	Odds ratio*	95% CI	Adjusted odds ratio*	95% CI
Age, per year	0.93	0.89, 0.96	0.89	0.82, 0.96
Gender				
Female	Reference	--	Reference	--
Male	0.65	0.41, 1.04	1.58	0.91, 2.74
Race				
White	Reference	--	Reference	--
Non-white	1.27	0.69, 2.34	0.85	0.42, 1.74
Clinical characteristics at index ED visit				
Rhythm diagnosis				
AF, any	Reference	--	Reference	--
AFL, isolated	3.18	1.38, 7.33	2.20	0.84, 5.77
AF/FL history				
Prior AF/FL diagnosis	Reference	--	Reference	--
New AF/FL diagnosis	2.93	1.81, 4.73	3.10	1.72, 5.58
Onset of symptoms				
Recent-onset (<48 hrs)	Reference	--	Reference	--
Not recent (≥48 hrs)	2.06	1.15, 3.69	2.31	1.03, 5.21
Unclear	1.54	0.91, 2.62	1.10	0.54, 2.23
AF/AFL categorization				
Chronic/unremitting	Reference	--	Reference	--
Intermittent/recurrent	2.61	1.19, 5.74	4.56	1.65, 12.60
Unclear	3.44	1.42, 8.38	3.43	1.14, 10.34
ED cardiologist consultation				
No	Reference	--	Reference	--
Yes	2.47	1.54, 3.96	1.89	1.10,3.23
ED discharge rhythm				
Sinus rhythm	Reference	--	Reference	--
AF/FL	1.67	1.05, 2.64	2.65	1.35, 5.21
ATRIA stroke risk				
Score, per point increase above 6	0.90	0.84, 0.96	1.10	0.96, 1.26
HAS-BLED hemorrhage risk score				
Score, per point increase	0.86	0.73, 1.02		
Categorical				
Low risk (<3)	Reference	--		
High risk (≥3)	0.59	0.34, 1.01		

*ED*, emergency department; *AF*, atrial fibrillation; *AFL*, atrial flutter; *AF/FL*, atrial fibrillation or flutter; *ATRIA*, Anticoagulation and Risk Factors in Atrial Fibrillation; *HAS-BLED*, Hypertension, Abnormal renal/liver function, Stroke, Bleeding history or predisposition, Labile international normalized ratio, Elderly (>65 years), Drugs or alcohol; *CI*, confidence interval.

* Reference group includes individuals with no anticoagulation initiation by 30 days after the index ED visit.

**Table 4:** Documentation of reasons for withholding anticoagulation for high-risk patients with atrial fibrillation and flutter discharged home from the emergency department (n=227).

	N (%)
Reasons for withholding anticoagulation	
Not documented	88 (38.8)
Documented	139 (61.2)
Physician concerns*	86 (37.9)
Bleed risk (including fall risk)	29 (12.8)†
Defer decision to outpatient physician	23 (10.1)
Restoration of sinus rhythm has reduced risk	19 (8.4)
Assents with another physician’s recommendation (either in prior notes or during consultation)	15 (6.6)
Perceived to be low risk for stroke, independent of sinus rhythm	6 (2.6)
Already on LMWH or non-aspirin antiplatelet agent	3 (1.3)
Patient concerns*	60 (43.2)
Prefers to discuss further with outpatient provider	20 (8.8)
Declines anticoagulation, no explanation documented	20 (8.8)
Previously discontinued‡	10 (4.4)
Perceived bleed risk	7 (3.0)
Frequent phlebotomy required	3 (1.3)

*LMWH*, low molecular weight heparin.

* Percentage calculated from cases in which the reason for withholding anticoagulation was documented (n=139). Seven cases included documentation of both physician and patient concerns.

† In 15 of these 29 cases the physician specified that their concern was the risk of falling.

‡ Reasons for previous discontinuation of warfarin were documented in six cases and included intolerance (n=2), bleeding or easy bruising (n=2), allergy (n=1), and non-adherence (n=1).
