# Supplementary material for: Thromboprophylaxis for Patients with High-risk Atrial Fibrillation and Flutter Discharged from the Emergency Department
Source: West J Emerg Med. 2018 Feb 12;19(2):346–60. doi: 10.5811/westjem.2017.9.35671 (PMC5851510; doi:10.5811/westjem.2017.9.35671)
Supplement: Supplementary file 1 [file wjem-19-346-s001.docx]

**Thromboprophylaxis for Patients with High-risk Atrial Fibrillation and Flutter**

**Discharged from the Emergency Department**

**Appendix**

**Table A1.** HAS-BLED variable definitions from their original study^1^ and revised for use in the TAFFY study

| **Risk Factor (points)** | **Original Definition** | **Revised Definition for TAFFY Study** |
| --- | --- | --- |
| **H**ypertension (1) | SBP >160 mm Hg | ≥1 outpatient visit with SBP >160 mm Hg in year before index date |
| **A**bnormal renal and liver function (1 each) | Chronic dialysis, renal transplantation, serum creatinine ≥200 μmol/L (renal) or chronic hepatic disease or abnormal bilirubin, alkaline phosphatase, alanine aminotransferase, aspartate aminotransferase (liver) | Chronic dialysis, renal transplantation (ever) or eGFR<45 in 1 year prior to index visit (renal).  Chronic hepatic disease diagnosis in year prior to index, liver transplantation (ever), or abnormal bilirubin, alkaline phosphatase, alanine aminotransferase, or aspartate aminotransferase in 3 months prior to index visit (liver) |
| **S**troke (1) | Previous history, particularly lacunar | Same |
| **B**leeding (1) | Bleeding history (major bleed) or predisposition (anemia) | Major bleed event (including hemorrhagic stroke) in prior year, or anemia diagnosis or low hemoglobin (<13 g/dL in men and <12 g/dL in women) in 90 days prior to index visit |
| **L**abile INRs (1) | Out of range >60% of the year prior to index | Same |
| **E**lderly (1) | Age >65 years | Same |
| **D**rugs or Alcohol (1 each) | Antiplatelet agents, non-steroidal anti-inflammatory drugs, excess alcohol | Evidence of antiplatelet prescription fills from pharmacy records in 6 months prior to index, or aspirin or antiplatelet medications on medication review list at the index visit (drug). Diagnosis of alcohol dependence or abuse in the year prior to the index visit (alcohol). |

eGFR, estimated glomerular filtration rate; INR, international normalized ratio; SBP, systolic blood pressure; TAFFY, Treatment of Atrial Fibrillation and Flutter in the emergencY department.

1. Pisters R, Lane DA, Nieuwlaat R, de Vos CB, Crijns HJ, Lip GY. A novel user-friendly score (HAS-BLED) to assess 1-year risk of major bleeding in patients with atrial fibrillation: the Euro Heart Survey. *Chest*. 2010;138:1093-1100.

**Table A2.** Characteristics of emergency department (ED) patients with atrial fibrillation or flutter enrolled and not enrolled in the TAFFY study

|  | **Enrolled patients**  **N=1,980 (69.5%)** | **Unenrolled patients**  **N=869 (30.5%)** | **P value*** |
| --- | --- | --- | --- |
| **Patient Characteristics** |  |  |  |
| **Age at ED Visit** |  |  |  |
| Age (yr), mean (SD) | 70.6 (14.0) | 71.3 (13.9) | 0.18 |
| Age category (yr), n (%) |  |  | 0.08 |
| <45 | 79 (4.0) | 38 (4.4) |  |
| 45 to 64 | 540 (27.3) | 198 (22.8) |  |
| 65 to 74 | 497 (25.1) | 240 (27.6) |  |
| ≥75 | 864 (43.6) | 393 (45.2) |  |
| Female gender, n (%) | 1004 (50.7) | 425 (48.9) | 0.38 |
| **Race/ethnicity, n (%)** |  |  | 0.47 |
| White/European | 1590 (80.3) | 685 (78.8) |  |
| Black/African American | 167 (8.4) | 90 (10.4) |  |
| Asian/Pacific Islander | 146 (7.4) | 66 (7.6) |  |
| Native Hawaiian/Other Pacific  Islander | 16 (0.8) | 7 (0.8) |  |
| Other/Unknown | 61 (3.1) | 21 (2.4) |  |
| **Comorbidities, n (%)** |  |  |  |
| History of atrial fibrillation or flutter | 1009 (51.0) | 392 (45.1) | <0.01 |
| Hypertension | 1,449 (73.2) | 647 (74.5) | 0.48 |
| Proteinuria | 945 (47.7) | 427 (49.1) | 0.49 |
| Coronary heart disease | 432 (21.8) | 199 (22.9) | 0.52 |
| Diabetes mellitus | 421 (21.3) | 194 (22.3) | 0.53 |
| Chronic heart failure | 326 (16.5) | 162 (18.6) | 0.16 |
| eGFR<45 ml/min/1.73 m2 or end-stage renal disease | 326 (16.5) | 151 (17.4) | 0.55 |
| Peripheral artery disease | 90 (4.6) | 44 (5.1) | 0.55 |
| Stroke | 21 (1.1) | 13 (1.5) | 0.33 |
| **ATRIA Study stroke risk score** |  |  |  |
| Mean (SD) | 8.2 (6.0) | 8.7 (6.1) | 0.09 |
| Low risk (≤5) | 772 (39.0) | 309 (35.6) | 0.19 |
| Moderate risk (6) | 153 (7.7) | 66 (7.6) |  |
| High risk (≥7) | 1,055 (53.3) | 494 (56.9) |  |
| **HAS-BLED hemorrhage risk score** |  |  |  |
| Mean (SD) | 2.1 (1.5) | 2.1 (1.5) | 0.76 |
| Categorical, n (%) |  |  |  |
| Low risk (<3) | 1293 (65.3) | 546 (62.8) | 0.20 |
| High risk (≥3) | 687 (34.7) | 323 (37.2) |  |

TAFFY, Treatment of Atrial Fibrillation/Flutter in emergencY medicine; ATRIA, Anticoagulation and Risk Factors in Atrial Fibrillation; HAS-BLED, Hypertension, Abnormal renal/liver function, Stroke, Bleeding history or predisposition, Labile international normalized ratio, Elderly (> 65 years), Drugs/alcohol concomitantly.

* Reported P values are from likelihood ratio chi-square tests for categorical comparisons. P values for mean comparisons are from Student T-tests for 2 group comparisons.
